# Early Bronchiolitis Contributes to Preschool Asthma

**DOI:** 10.3390/children8121176

**Published:** 2021-12-13

**Authors:** Jih-Chin Chang, Jeng-Yuan Chiou, Jiunn-Liang Ko, Jing-Yang Huang, Ko-Huang Lue

**Affiliations:** 1 Department of Pediatrics, Chang Bing Show Chwan Memorial Hospital, Changhua 505, Taiwan; sunsquare123@gmail.com; 2 Institute of Medicine, Chung Shan Medical University, Taichung 402, Taiwan; jlko@csmu.edu.tw (J.-L.K.); wchinyang@gmail.com (J.-Y.H.); 3 School of Health Policy and Management, Chung Shan Medical University, Taichung 402, Taiwan; tom@csmu.edu.tw; 4 Department of Pediatrics, Chung Shan Medical University Hospital, Taichung 402, Taiwan; 5 School of Medicine, Chung Shan Medical University, Taichung 402, Taiwan

**Keywords:** infant, bronchiolitis, hospitalization, asthma, atopic dermatitis

## Abstract

This study aims to analyze whether bronchiolitis in children younger than one-year-old contributes to subsequent asthma. Medical data were retrieved from the National Health Insurance Research Database of Taiwan. Participants were divided into study (N = 65,559) and control (N = 49,656) groups, depending on whether they had early bronchiolitis. Incidences of asthma, potential comorbidities, and associated medical conditions were compared. The incidence of childhood asthma was significantly higher in the study group (aHR = 1.127, 95% CI: 1.063–1.195). Children with bronchiolitis hospitalization displayed higher asthma risk in the period between two and four years of age. The risk diminished as the children grew up. No relevant synergistic effects were found between bronchiolitis and atopic dermatitis. In conclusion, bronchiolitis before one year of age exhibits predictive value for development of preschool asthma, especially in children with bronchiolitis hospitalizations.

## 1. Introduction

Acute bronchiolitis is a common cause of hospitalization in young children and usually presents with cough, wheezing, and dyspnea [1,2]. Most patients recover with supportive care, but recurrent wheezing may become a troubling problem in some children [3].

Pediatric atopic diseases include atopic dermatitis, allergic rhinitis, asthma, and others. These diseases are important childhood health issues and influence the daily life of children to variable degrees. Among them, asthma is a severe hyperreactive airway disease. Acute asthma attacks cause a wheezy cough and shortness of breath, requiring timely medical treatments such as bronchodilators and steroids. The wheezing and dyspnea observed in asthma resemble the symptoms of bronchiolitis, and several studies have investigated the relationship between these two diseases [3,4].

Viral bronchiolitis is the main cause of wheezing during infancy. Studies have shown that some cases of infantile wheezing may progress to asthma [5]. Several factors such as recurrent wheezing, male sex, atopic diseases, and specific pathogens have been reported to contribute to asthma development in children with bronchiolitis [6,7,8,9]. However, there are currently no definitive conclusions about this causality [3]. We hypothesized that children with early bronchiolitis have a higher risk of subsequent asthma. The present study aimed to analyze the relationship between bronchiolitis before one year of age and subsequent childhood asthma, as well as the influence of associated determinants.

## 2. Materials and Methods

### 2.1. Databases

The National Health Insurance (NHI) program was established in Taiwan in 1995 and covers more than 99.5% of the Taiwanese population. The National Health Insurance Research Database (NHIRD) includes comprehensive insurance-claims data from the NHI program and provides reliable data for population-based disease research. All the insurance claims in the NHI program must be reviewed and audited by medical reimbursement specialists, upholding the validity and accuracy of the datasets. The Longitudinal Health Insurance Database (LHID), managed by the Taiwan National Health Research Institute (NHRI), allows researchers to track longitudinal changes related to medical utilization [10]. It contains aggregated secondary data without personal identification and has been used extensively in various medical research fields [4,11,12]. These datasets are trustworthy resources for analyzing population-based relationships between, and determinants of, bronchiolitis and childhood asthma.

### 2.2. Study Population and Design

We used the LHID 2010 containing data processed by the NHRI between 2001 and 2010 for our analysis. The LHID 2010 contains claims data of a subset containing 1 million beneficiaries, randomly sampled from the NHIRD’s 2010 beneficiary registry. There are no significant differences in sex distribution, age distribution, or average insured payroll-related amount between the LHID 2010 and NHIRD [13]. This study was approved by the Institutional Review Board of Chang Bing Show Chwan Memorial Hospital (project identification code: SCMH_IRB Number: 1051109; date of approval: 7 December 2016).

**Figure 1:**
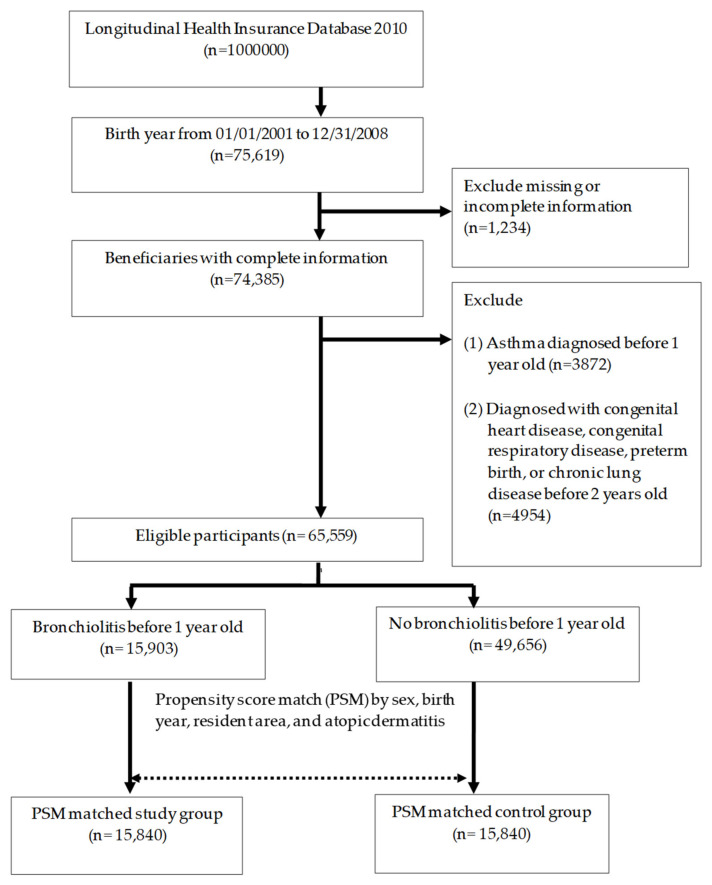
Flow chart of case enrollment from the National Health Insurance Research Database in Taiwan.

Information regarding children born from 2001 to 2008 was retrieved. We excluded patients with asthma diagnosed before the age of 1 years (identified by International Classification of Diseases, Ninth Revision, Clinical Modification (ICD-9-CM): 493), and patients with co-morbidities including preterm birth (ICD-9-CM: 765), congenital heart disease (ICD-9-CM: 745, 746, 747), congenital respiratory disease (ICD-9-CM: 748), or chronic lung disease (ICD-9-CM: 770.7). The remaining patients were enrolled in either the study or the control group according to bronchiolitis diagnosis (ICD-9-CM: 466.1) before one year of age (Figure 1). The records of each patient, as well as associated conditions including atopic dermatitis (ICD-9-CM: 691.8), outpatient visits, and admissions, were retrieved and compared among groups analyzing the influences of early bronchiolitis, and the risk interactions between bronchiolitis and other determinants.

In Taiwan, clinicians diagnose asthma based on Global Initiative for Asthma (GINA) guidelines. Children with asthma should have positive data in the chart records describing past history, drug history, family history, atopy history, allergen-specific test, lung function test, or asthma control score. In the present study, asthma diagnoses were ascertained from records of either admission datasets (at least one admission with discharge diagnosis of asthma) or outpatient datasets. Diagnoses of asthma in outpatient datasets were included only if the patient had at least 3 visits due to asthma and had used one of the following anti-asthmatic medicines within 6 months after diagnosis: adrenergics (Anatomical Therapeutic Chemical (ATC) codes: R03A, R03C), xanthines (ATC R03DA), or steroids/β2 agonists (ATC R03BA, R03AK06, and R03AK07).

### 2.3. Controls and Propensity Score Matching

We used propensity score matching (PSM) [14] to control for potential confounding factors such as sex distribution, age distribution, geographic region, atopic dermatitis (ICD-9-CM: 691.8) and general medical service uses. We created 1:1 propensity score-matched controls as follows: first we constructed a logistic regression and set the dependent variable (bronchiolitis) and the independent variables (sex, birth year, residence area, atopic dermatitis and medical service use). Then, we found an optimal model using stepwise selection and calculated the propensity score for each patient.

### 2.4. Statistical Analysis

Data analysis was performed using SAS software (version 9.4; SAS Institute, Cary, NC, USA). We calculated the person-years of follow-up from the age of 2 years until patient withdrawal, asthma diagnosis, or the end of the study. Incidence rates with 95% confidence intervals (C.I.) were defined as cases per 1000 person-years and estimated by Poisson regression. Kaplan–Meier curves were generated; the log-rank test was used to test differences in cumulative asthma risk among groups. Multivariate Cox proportional hazards regression was performed to estimate the hazard ratios (HR) and 95% confidence intervals (CI) of asthma due to bronchiolitis exposure. To explore risk interactions between bronchiolitis and sex or atopic dermatitis with regard to asthma risk, we conducted a stratified analysis and tested the interaction term in a mixed model. A *p*-value lower than 0.05 was considered statistically significant.

## 3. Results

In total, 65,559 children were included in the analysis, comprising 15,903 study group patients and 49,656 controls (Figure 1). After PSM, 15,840 patients remained in the study group and 15,840 patients in the control group. No significant differences were found between the groups regarding birth year, sex, health insurance district, medical service uses, and comorbidity of atopic dermatitis (Table 1).

As seen in Table 2, 2362 patients (14.9%) in the bronchiolitis group were diagnosed with asthma after two year of age, and the risk was significantly higher than the control group (HR = 1.127, 95% C.I: 1.063–1.195).

**Figure 2:**
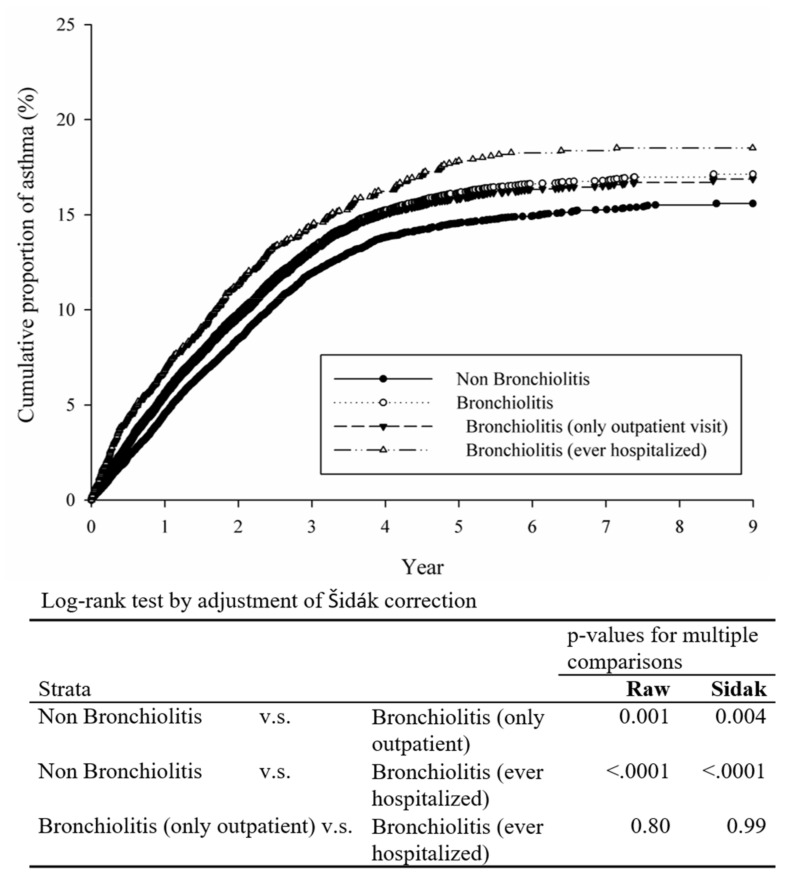
Cumulative incidence rate of asthma stratified by study groups.

To further analyze the influence of bronchiolitis visits and hospitalization on asthma development, we divided patients into several subgroups. The incidence of asthma was higher in the subgroup with bronchiolitis admissions, and the control group had the lowest incidence of asthma. We also observed an increasing trend regarding the hazard ratios of childhood asthma in the subgroups ever hospitalized for bronchiolitis (Table 2). There was a similar pattern in the cumulative probabilities of asthma incidence among the study subgroups (Figure 2). Patients without any bronchiolitis had the lowest cumulative incidence of asthma, and patients in the subgroup ever hospitalized for bronchiolitis had the highest cumulative incidence of asthma.

Most cases of childhood asthma were first diagnosed at an age of 2–4 years (83.4% in the control group and 84% in the bronchiolitis group). Furthermore, the adjusted hazard ratio of developing asthma in patients with early bronchiolitis was significantly higher at an age of 2–4 years but not at ages of 5–7 years or >7 years (Table 3).

Subgroup analyses revealed risk interactions between bronchiolitis and specific conditions regarding asthma development risk (Table 4). Effect of bronchiolitis on asthma development risk was modified by atopic dermatitis with borderline significance (interaction *p* = 0.0499). Further analyses of risk interactions showed that the hazard ratios of childhood asthma were significantly higher in patients without atopic dermatitis who had ever been hospitalized for bronchiolitis (HR = 1.324, 95% C.I: 1.172–1.496). On the other hand, no significant interaction was revealed between sex and bronchiolitis regarding asthma development (interaction *p* = 0.1047). However, the hazard ratios seemed to be higher in male patients with bronchiolitis events.

## 4. Discussion

Our study used longitudinal, population-based data to analyze the relationship between bronchiolitis and childhood asthma. We found an increased risk of childhood asthma in children diagnosed with bronchiolitis before the age of one year. We also demonstrated that bronchiolitis hospitalization contributed to higher risk of subsequent childhood asthma than bronchiolitis outpatient visits. Furthermore, the risk of asthma at an age of 2–4 years was higher than that at an age of ≥5 years. The effects of atopic dermatitis and sex were investigated in the subgroup analysis, and we found the risk of developing asthma was significantly modulated by early bronchiolitis events in patients without atopic dermatitis.

Wheezing is common in infants and toddlers. Most children outgrow their symptoms, but sometimes wheezing persists into adolescence or even adulthood [5]. The relationship between infantile wheeze and childhood asthma has been often investigated but remains unclear. 

Viral bronchiolitis caused by pathogens, including respiratory syncytial virus, human rhinovirus, human metapneumovirus, and influenza [15,16], is the leading cause of infantile wheezing. The first two viruses are the most common and have been reported to be related to the development of asthma [16,17]. Studies from Taiwan have indicated that respiratory syncytial virus is the leading pathogen causing bronchiolitis, and bronchiolitis occurs year-round with two slight peaks in spring and fall [15,18]. This is different from reports from North America and Europe, which have shown a predominant peak in winter [1,2]. Although we cannot accurately determine the actual pathogens causing bronchiolitis from our database search, the results of our study support that bronchiolitis at a young age strongly correlates with childhood asthma, as numerous published studies have reported [4,19,20] (Table 2).

To further explore the relationship between wheezing episodes and childhood asthma, we analyzed both bronchiolitis outpatient visits and hospital admissions (Table 2). We discovered that bronchiolitis hospitalization was more influential than outpatient visits regarding asthma risk. Our results further indicated that, as admissions increased, the risk of asthma seemed to get higher. Our study revealed the severity-dependent relationship between early bronchiolitis and subsequent childhood asthma, and it supports previous study reports [21,22].

Furthermore, bronchiolitis before one year old was significantly related to increased asthma risk in preschoolers but not among older children (Table 3). Debates have existed about the relationship between bronchiolitis and asthma in later life [7,23], and our findings support the idea that infection-associated respiratory morbidities could diminish in older children, who have more robust and larger airways [4,23]. 

As for the causality between bronchiolitis and asthma, some possibilities have been proposed. Children with asthmatic characteristics may have susceptibility and more easier contract bronchiolitis [8]. On the other hand, bronchiolitis or early-life infections have been shown to influence the airway development, and further increase the risk of developing future asthma [19]. The two mechanisms may coexist [3]. The present analysis limited the bronchiolitis diagnosed before one-year-old to eliminate the children with possible asthmatic characteristics, and the risk of asthma remained higher in the children with early bronchiolitis group (Table 2). 

Atopic dermatitis is one of the most common allergic diseases occurring during infancy, often observed as the first step of the atopic march [24]. It has been reported that the presence of recurrent wheeze in combination with allergic diseases and parental atopy helps in predicting later asthma [22]. Children with atopy seem to be prone to bronchiolitis, but relationships between bronchiolitis and allergic sensitization are often debated [7,8,17,25,26,27]. In our study, an interaction was demonstrated between atopic dermatitis and infant bronchiolitis regarding asthma risk (Table 4). The effect of early bronchiolitis on later asthma risk seemed to be more pronounced within subgroups without atopic dermatitis. Our study suggests no relevant synergistic effects among these diseases. Nevertheless, this phenomenon should be carefully interpreted for borderline significance, and the mechanism by which atopic sensitization interferes with bronchiolitis in asthma development still warrants further investigation.

Mikalsen et al. [25] reported that bronchial hyper-responsiveness and asthma after early bronchiolitis occurs mostly in boys. Some reports have also demonstrated that young bronchiolitis and asthma sufferers are mainly male [9]; however, Lin et al. [20] reported that girls with bronchiolitis were slightly more at risk of developing asthma than boys with bronchiolitis. Although our results revealed increased asthma risk from early bronchiolitis in the male subgroup, no significant interaction was observed between sex and bronchiolitis with regard to asthma risk (Table 4). Despite the sex-related disparities in asthma [9,28], our study indicated that the sex does not modulate the effect of early bronchiolitis on later asthma development.

Some diseases during infancy have been reported as related to severe bronchiolitis and even later asthma, such as premature birth, congenital heart disease, and chronic lung disease [29]. However, data relating to these conditions were incomplete in the present study’s database search, and comprehensive analysis of their influences on childhood asthma in children with bronchiolitis could not be performed. Thus, we excluded patients with these conditions from our analysis in advance, and our results should generally be considered to apply to otherwise healthy children.

The present study, like previous investigations, suggests that early bronchiolitis is strongly related to childhood asthma, and we were able to add to the knowledge of the influence of bronchiolitis severity, the risk distribution by age, and associated conditions, such as atopic dermatitis and sex differences, on asthma development. However, asthma is a chronic, complicated, and multi-factorial disease [30]. The study is limited in that the laboratory data and patient histories were not included in insurance claims data from the NHIRD. We also lacked sufficient data to completely evaluate all factors involved, such as genetic and environmental influences.

Children with early bronchiolitis before one year of age have an increased risk of developing childhood asthma, especially among preschoolers. The risk seems to be diminished as the children grow up. In addition, the factors about bronchiolitis severity, such as repeated admissions, increase the risk of later asthma. The effect of early bronchiolitis on later asthma risk may be more apparent in children without atopic dermatitis and should be carefully evaluated. Parents of children with early bronchiolitis should be informed of the higher risk of later asthma and should seek medical assistance upon the first signs of asthma.

## 5. Conclusions

This study demonstrates that bronchiolitis before one year of age exhibits predictive value for development of preschool asthma, especially in children with bronchiolitis hospitalizations.

## Figures and Tables

**Table 1:** Characteristics of the study and the control groups.

Early Bronchiolitis			
	No (N = 15,840)	Yes (N = 15,840)	*p*-Value
Birth year			0.86
2001	2469 (15.59%)	2413 (15.23%)	
2002	2228 (14.07%)	2191 (13.83%)	
2003	1929 (12.18%)	1971 (12.44%)	
2004	2261 (14.27%)	2220 (14.02%)	
2005	1822 (11.5%)	1824 (11.52%)	
2006	1808 (11.41%)	1876 (11.84%)	
2007	1729 (10.92%)	1753 (11.07%)	
2008	1594 (10.06%)	1592 (10.05%)	
Sex			0.84
Female	6874 (43.4%)	6857 (43.29%)	
Male	8966 (56.6%)	8983 (56.71%)	
Division			0.20
Taipei Division	5105 (32.23%)	5047 (31.86%)	
Northern Division	2748 (17.35%)	2741 (17.30%)	
Central Division	3620 (22.85%)	3587 (22.65%)	
Southern Division	2118 (13.37%)	2138 (13.50%)	
Koping Division	2023 (12.77%)	2043 (12.90%)	
Eastern Division	226 (1.43%)	284 (1.79%)	
Atopic dermatitis before 2 y/o			0.42
No	12822 (80.95%)	12766 (80.59%)	
Yes	3018 (19.05%)	3074 (19.41%)	
Emergency care times before 2 y/o			0.52
0	5966 (37.66%)	6062 (38.27%)	
1	3807 (24.03%)	3755 (23.71%)	
≥2	6067 (38.30%)	6023 (38.02%)	
Dentistry before 2 y/o			0.003
0	15028 (94.87%)	14908 (94.12%)	
≥1	812 (5.13%)	932 (5.88%)	
Traditional Chinese medicine before 2 y/o			0.029
0	13778 (86.98%)	13646 (86.15%)	
≥1	2062 (13.02%)	2194 (13.85%)	
Preventive Health Care before 2 y/o			0.07
<5	6718 (42.41%)	6875 (43.40%)	
≥5	9122 (57.59%)	8965 (56.60%)	
Hospitalization days before 2 y/o			0.24
0	8731 (55.12%)	8729 (55.11%)	
1–2	4189 (26.45%)	4091 (25.83%)	
≥3	2920 (18.43%)	3020 (19.07%)	

Data are presented as frequencies (*n*) and percentages.

**Table 2:** Asthma incidence rate in groups with or without bronchiolitis (per 10^3^ person years).

			Risk of Asthma after 2 Years Old			
	*n*	Follow up Person Year	Event	Incidence (per 10^3^ Person Years)	Crude HR (95%CI)	Adjusted HR (95%CI)
No Bronchiolitis	15,840	75,591.56	2128	28.15 (26.98–29.37)	Reference	Reference
Bronchiolitis	15,840	74,085.85	2362	31.88 (30.62–33.19)	1.12 (1.06–1.19)	1.12 (1.06–1.19)
Bronchiolitis (without hospitalization)	13,333	62,646.41	1955	31.21 (29.85–32.62)	1.10 (1.03–1.17)	1.10 (1.04–1.17)
Bronchiolitis (hospitalization for 1–2 times)	2454	11,182.35	397	35.50 (32.17–39.17)	1.24 (1.11–1.38)	1.21 (1.09–1.35)
Bronchiolitis (hospitalization more than 3 times)	53	257.10	10	38.89 (20.93–72.28)	1.41 (0.75–2.62)	1.28 (0.69–2.39)

The adjusted hazard ratio was estimated by multiple Cox regression, the covariates included birth year, sex, resident region, comorbidity with atopic dermatitis, emergency visit, dental visit, Chinese medicine visit, preventive health care, and length of hospital stay before 2 years old.

**Table 3:** Risk of asthma stratified by follow up time.

	aHR (95% C.I.) of Asthma at Specific Follow up Time Interval		
	2–4 Years Old	5–7 Years Old	>7 Years Old
Groups			
No Bronchiolitis	Reference	Reference	Reference
Bronchiolitis (OPD care, without hospitalization)	1.14 (1.06–1.24)	1.04 (0.94–1.16)	1.04 (0.71–1.51)
Bronchiolitis (with hospitalization)	1.32 (1.16–1.50)	1.06 (0.87–1.28)	0.81 (0.37–1.78)

Abbreviations: aHR, adjusted hazard ratio; C.I., confidence interval; OPD, outpatient department. The adjusted hazard ratio was estimated by multiple Cox regression, the covariates included birth year, sex, resident region, comorbidity with atopic dermatitis, emergency visit, dental visit, Chinese medicine visit, preventive health care, and length of hospital stay before 2 years old.

**Table 4:** Risk of Bronchiolitis patients on asthma by specific sub-groups.

	aHR (95% C.I.)		
	No Bronchiolitis	Bronchiolitis (OPD Care, without Hospitalization)	Bronchiolitis (with Hospitalization)
Sex			
Female	Reference	1.09 (0.99–1.21)	1.03 (0.86–1.24)
Male	Reference	1.11 (1.02–1.20)	1.33 (1.16–1.51)
*p* for interaction	0.10		
Atopic dermatitis before 2 years old			
With Atopic dermatitis	Reference	1.07 (0.95–1.21)	0.95 (0.76–1.18)
Without Atopic dermatitis	Reference	1.11 (1.04–1.20)	1.32 (1.17–1.49)
*p* for interaction	0.049		

Abbreviations: aHR, adjusted hazard ratio; C.I., confidence interval; OPD, outpatient department. The adjusted hazard ratio was estimated by multiple Cox regression, the covariates included birth year, sex, resident region, comorbidity with atopic dermatitis, emergency visit, dental visit, Chinese medicine visit, preventive health care, and length of hospital stay before 2 years old.

## Data Availability

The data presented in this study are available on request from the corresponding author. The data are not publicly available due to restrictions e.g., privacy or ethical.
